# Using a nomogram based on the controlling nutritional status score to predict prognosis after surgery in patients with resectable gastric cancer

**DOI:** 10.1186/s12876-025-03766-6

**Published:** 2025-03-17

**Authors:** Xinghao Ma, Xiaoyang Jiang, Hao Guo, Jiajia Wang, Tingting Wang, Jiahu Yao, Song Liang, Xiuming Lu, Chuanxia Wang, Chuansi Wang

**Affiliations:** 1 Department of Clinical Nutrition, Lu’an Hospital, Anhui Medical University, NO. 21 of Wanxi West Road, Jin’an District, Lu’an, 237005 China; 2 Department of Nutrition, School of Public Health, Anhui Medical University, NO. 81 of Meishan Road, Shushan District, Hefei, 230032 China; 3 Department of General Surgery, Lu’an Hospital, Anhui Medical University, NO. 21 of Wanxi West Road, Jin’an District, Lu’an, 237005 China

**Keywords:** CONUT, Gastric cancer, Surgery, Prognosis, Nomogram

## Abstract

**Background:**

Various studies have shown that the controlling nutritional status (CONUT) score contributes to assessing the prognosis of cancer patients. This study aimed to establish a nomogram based on the CONUT score and several other important parameters based on patient age and tumor characteristics to accurately forecast the overall survival (OS) of patients with resectable gastric cancer (GC).

**Methods:**

This study retrospectively recruited 404 individuals who received a potentially curative radical gastrectomy performed by the same group of surgeons at our medical center from January 2019 to December 2021. We used Cox regression analysis to identify independent prognostic factors influencing patients' OS. We establish a nomogram based on the outcomes of the multivariate analysis to forecast the 1, 2, and 3-year OS of GC patients.

**Results:**

Univariate Cox regression analysis revealed that the age, body mass index (BMI), hemoglobin (HGB), serum albumin (ALB), Serum carcinoembryonic antigen (CEA), CONUT score, tumor size, pT stage, pN stage, nerve invasion, vascular invasion, tumor differentiation, and postoperative chemotherapy were prognostic indicators of postoperative OS in GC patients (all *P* < 0.05). Multivariate Cox regression analysis indicated that the age (*P* = 0.015), CONUT score (*P* = 0.002), pT stage (T3 vs T1: *P* = 0.011, T4 vs T1: *P* = 0.026), pN stage (N2 vs N0: *P* = 0.002, N3 vs N0: *P* < 0.001), nerve invasion (*P* = 0.021) were the independent risk factors. The nomogram based on the CONUT score, with a C-index of 0.792, enhanced the predictive ability of the TNM staging system alone, which had a C-index of 0.718 for OS.

**Conclusion:**

The CONUT score can independently predict the OS for individuals with GC following surgery. The nomogram based on the CONUT score is a reliable tool for forecasting the postoperative survival of individuals with GC and may identify those patients wholesale benefit from a more aggressive treatment protocol.

## Introduction

Gastric cancer (GC) has become a substantial public health problem due to its high incidence and mortality rates globally. In 2020, there were over a million new cases of GC and roughly 769000 deaths from GC [1]. Patients with resectable GC are currently treated mainly through surgery. The prognosis of GC patients varies, even after radical resection, for various reasons, including age, malnutrition, tumor stage, lymph node metastases, and postoperative complications [2].

Currently, clinicians primarily use the 8th version of the American Joint Committee on Cancer tumor node metastasis (AJCC-TNM) staging system to estimate the prognosis for GC patients. The numerous factors that impact the prediction of GC result in considerable individual variability across individuals, and the TNM stage alone may have limited predictive utility. Consequently, looking into a more thorough, precise, and reliable assessment approach is crucial to predict the postoperative outcome of GC patients.

Numerous clinical studies have shown that host-specific factors, including preoperative nutritional status, systemic inflammation, and other tumor-related factors, impact GC patients' prognosis [3, 4]. The Controlling Nutritional Status (CONUT) score incorporates serum ALB, cholesterol levels, and lymphocyte count to indicate the patients' preoperative nutritional and immunological statuses. Several clinical studies have acknowledged its value as a validated and practical nutritional status evaluation for predicting short- and long-term prognosis following radical gastrectomy since its initial report in 2005 [5–9]. Although several studies report a statistical connection between the CONUT score and prognosis, the predictive power of the CONUT score for prognosis has yet to be numerically validated.

In this study, we established a nomogram model based on age, CONUT score, and tumor-associated characteristics to predict the individual survival of GC patients. We then compared it with the conventional AJCC-TNM stage to see if the nomogram could offer patients and their physicians more accurate prognostic assessments.

## Methods

### General data

The study's participants included 404 GC patients who received radical gastrectomy for GC by the same group of surgeons at our institution from January 2019 to December 2021. The criteria for inclusion were as follows: (1) GC Patients were verified by preoperative gastroscopic biopsy. (2) Radical gastrectomy performed. (3) Age over 18 years. (4) Patients having comprehensive clinical and pathological data. The following were the exclusion requirements: (1) Neoadjuvant chemotherapy before surgery; (2) Only R1/2 resection; (3) Gastric stump cancer; (4) Tumor with distant metastasis; (5) Palliative surgery. The current study has received the approval of our hospital's Ethics Committee.

### Data acquisition

Clinical and pathological information on the participants in this study was gathered and recorded from medical records. The data mainly included the following: age, sex, hypertension, diabetes mellitus (DM), chronic obstructive pulmonary disease (COPD), previous abdominal surgery, smoking, drinking, body mass index (BMI), tumor location, tumor size, Borrmann classification, pTNM stage, tumor differentiation which included signet cell cancer, nerve invasion, vascular invasion, preoperative total lymphocyte count (TLC), total cholesterol (TC), serum albumin (ALB), hemoglobin (HGB), preoperative carcinoembryonic antigen (CEA), carbohydrate antigen 199 (CA199), carbohydrate antigen 125 (CA125). CONUT scores were calculated according to the criteria for each indicator in Table 1.

**Table 1:** Calculation of CONUT score

Variables	Malnutrition status			
	**Normal**	**Light**	**Moderate**	**Severe**
ALB (g/dl)	≥ 3.5	3.0 ≤ ALB < 3.5	2.5 ≤ ALB < 3.0	< 2.5
Score	0	2	4	6
TC (mg/dl)	≥ 180	140 ≤ TC < 180	100 ≤ TC < 140	< 100
Score	0	1	2	3
TLC (mg/ml)	≥ 1600	1200 ≤ TLC < 1600	800 ≤ ALB < 1200	< 800
Score	0	1	2	3
Total score	0–1	2–4	5–8	9–12

### Follow-ups

Telephone follow-up and outpatient reviews served to monitor the enrolled patients until March 2023. Patients were followed every three months for the first two years following surgery. Patients were then followed every six months after that. Patients were evaluated with laboratory tests, physical examinations, and imaging. OS was outlined as the period from the operation date to the final follow-up visit or death for any reason.

### Statistical analysis

Statistical analyses were performed using X-tile (3.6.1), SPSS (26.0), and R (4.3.0). Standard clinical variables such as BMI, HGB, ALB, CEA, CA125, and CA199 are assigned cut-off values that are evaluated using established criteria. Additionally, cut-off values for age (60 years), CONUT score (3), and tumor size (4 cm) were delineated using X-tile software [10]. The chi-square test was applied to analyzing categorical variables. Cox proportional hazards regression models were utilized for the multivariate analysis. The Kaplan–Meier method was used to generate survival curves, and the log-rank test was employed to assess the disparities between the survival curves. The nomogram was constructed using the outcomes of the multivariate analysis. The calibration plot approach was applied to assess the nomogram's conformity. The receiver operating characteristic (ROC) curve and Harrell's concordance index (C-index) were utilized to evaluate the nomogram model's predictive performance. The utility was evaluated using decision curve analysis. *P* values less than 0.05 were regarded as statistically significant.

## Results

### Patient characteristics

In this study, 404 gastric cancer patients who received a radical gastrectomy participated. 110 (27.2%) were female, and 294 (72.8%) were male. The patients' ages varied from 39 to 88, with a median of 68. The interquartile range of the follow-up period was 18 to 38 months, with 28.0 being the median. At 1, 2, and 3 years, the overall survival rates were 86.8, 76.0, and 68.2%, respectively. Stages I, II, and III of pTNM existed in 108 (26.7%), 103 (25.5%), and 193 (47.8%) individuals, respectively. Forty-two patients (10.4%) had high differentiation, 69 patients (17.1%) had moderate differentiation, and 293 patients (72.5%) had poor or no differentiation. Table 2 lists all of the patient's specific clinical features.

**Table 2:** Patient's clinical characteristics

Clinical characteristics	All (*n* = 404)
Age (median, IQR)	68(63–74)
Sex (male/female, *n*)	294/110
Smoking	
No	336(83.2)
Yes	68(16.8)
Drinking	
No	340(84.2)
Yes	64(15.8)
Hypertension	
No	275(68.1)
Yes	129(31.9)
DM	
No	364(90.1)
Yes	40(9.9)
Tumor location	
Upper	183(45.3)
Middle	89(22.0)
Lower	132(32.7)
Tumor size (cm)	
≤ 4	204(50.5)
> 4	200(49.5)
Tumor differentiation	
Well	42(10.4)
Moderate	69(17.1)
Poor or no	293(72.5)
Nerve invasion	
No	214(53.0)
Yes	190(47.0)
Vascular invasion	
No	220(54.5)
Yes	184(45.5)
Postoperative chemotherapy	
No	63(15.6)
Yes	341(84.4)
pTNM stage	
I	108(26.7)
II	103(25.5)
III	193(47.8)
Serum CEA (ng/mL)	
≤ 5	307(76.0)
> 5	94(23.3)
Serum CA125 (U/mL)	
≤ 35	392(97.0)
> 35	9(2.2)
Serum CA199 (U/mL)	
≤ 37	346(85.6)
> 37	55(13.6)
Type of gastrectomy	
Subtotal	140(34.7)
Total	264(65.3)
CONUT score	
≤ 3	337(83.4)
> 3	67(16.6)

### Correlation between preoperative CONUT scores and clinical characteristics

According to the cut-off value of the CONUT score, 337 patients who scored less than or equal to 3 were allocated to the low CONUT score group, whereas 67 patients who scored more than three were placed in the high CONUT score group. Table 3 displays the connections between 404 patients' clinical traits and CONUT scores. Age, COPD, HGB, ALB, tumor size, nerve invasion, vascular invasion, and OS were all associated with the CONUT score (*P* < 0.05 for all). Sex, BMI, drinking, smoking, hypertension, DM, previous abdominal surgery, tumor location, tumor differentiation, serum CEA, CA125, and CA199 were all unrelated to the CONUT score.

**Table 3:** Correlation between preoperative CONUT score and clinical features of GC patients

Characteristics	All (*n* = 404)	CONUT score			
		≤ 3 (*n* = 337)	> 3 (*n* = 67)	*χ*^*2*^	*p*
Age(years)				10.452	0.001
≤ 60	83(20.5)	79(23.4)	4(6.0)		
> 60	321(79.5)	258(76.6)	63(94.0)		
Sex				0.949	0.330
Male	294(72.8)	242(71.8)	52(77.6)		
Female	110(27.2)	95(28.2)	15(22.4)		
Smoking				0.010	0.921
No	336(83.2)	280(83.1)	56(83.6)		
Yes	68(16.8)	57(16.9)	11(16.4)		
Drinking				0.258	0.612
No	340(84.2)	285(84.6)	55(82.1)		
Yes	64(15.8)	52(15.4)	12(17.9)		
Hypertension				1.071	0.301
No	275(68.1)	233(69.1)	42(62.7)		
Yes	129(31.9)	104(30.9)	25(37.3)		
DM				0.081	0.777
No	364(90.1)	303(89.9)	61(91.0)		
Yes	40(9.9)	34(10.1)	6(9.0)		
COPD				9.387	0.002
No	385(95.3)	326(96.7)	59(88.1)		
Yes	19(4.7)	11(3.3)	8(11.9)		
Previous abdominal surgery				0.109	0.741
No	343(84.9)	287(85.2)	56(83.6)		
Yes	61(15.1)	50(14.8)	11(16.4)		
BMI (kg/m^2^)				4.137	0.126
< 18.5	55(13.6)	43(12.8)	12(17.9)		
18.5–23.9	245(60.6)	201(59.6)	44(65.7)		
≥ 24.0	104(25.7)	93(27.6)	11(16.4)		
HGB (g/L)				48.670	< 0.001
< 110/120	165(40.8)	112(33.2)	53(79.1)		
≥ 110/120	239(59.2)	225(66.8)	14(20.9)		
ALB (g/L)				162.661	< 0.001
< 35	44(10.9)	7(2.1)	37(55.2)		
≥ 35	360(89.1)	330(97.9)	30(44.8)		
Tumor location				1.615	0.446
Upper	183(45.3)	154(45.7)	29(43.3)		
Middle	89(22.0)	77(22.8)	12(17.9)		
Lower	132(32.7)	106(31.5)	26(38.8)		
Tumor size(cm)				6.917	0.009
≤ 4	204(50.5)	180(53.4)	24(35.8)		
> 4	200(49.5)	157(46.6)	43(64.2)		
Nerve invasion				5.177	0.023
No	214(53.0)	187(55.5)	27(40.3)		
Yes	190(47.0)	150(44.5)	40(59.7)		
Vascular invasion				4.042	0.044
No	220(54.5)	191(56.7)	29(43.3)		
Yes	184(45.5)	146(43.3)	38(56.7)		
Tumor differentiation				1.816	0.403
Well	42(10.4)	38(11.3)	4(6.0)		
Moderate	69(17.1)	56(16.6)	13(19.4)		
Poor or no	293(72.5)	243(72.1)	50(74.6)		
Serum CEA (ng/mL)				0.028	0.867
≤ 5	307(76.8)	257(76.7)	50(75.8)		
> 5	94(23.4)	78(23.3)	16(24.2)		
Serum CA125 (U/mL)				1.907	0.167
≤ 35	392(97.8)	329(98.2)	63(95.5)		
> 35	9(2.2)	6(1.8)	3(4.5)		
Serum CA199 (U/mL)				1.332	0.249
≤ 37	346(86.3)	292(87.2)	54(81.8)		
> 37	55(13.7)	43(12.8)	12(18.2)		
OS				18.525	< 0.001
Alive	277(68.6)	246(73.0)	31(46.3)		
Death	127(31.4)	91(27.0)	36(53.7)		

### Analysis of prognostic risk factors for GC patients after surgery

**Fig. 1:**
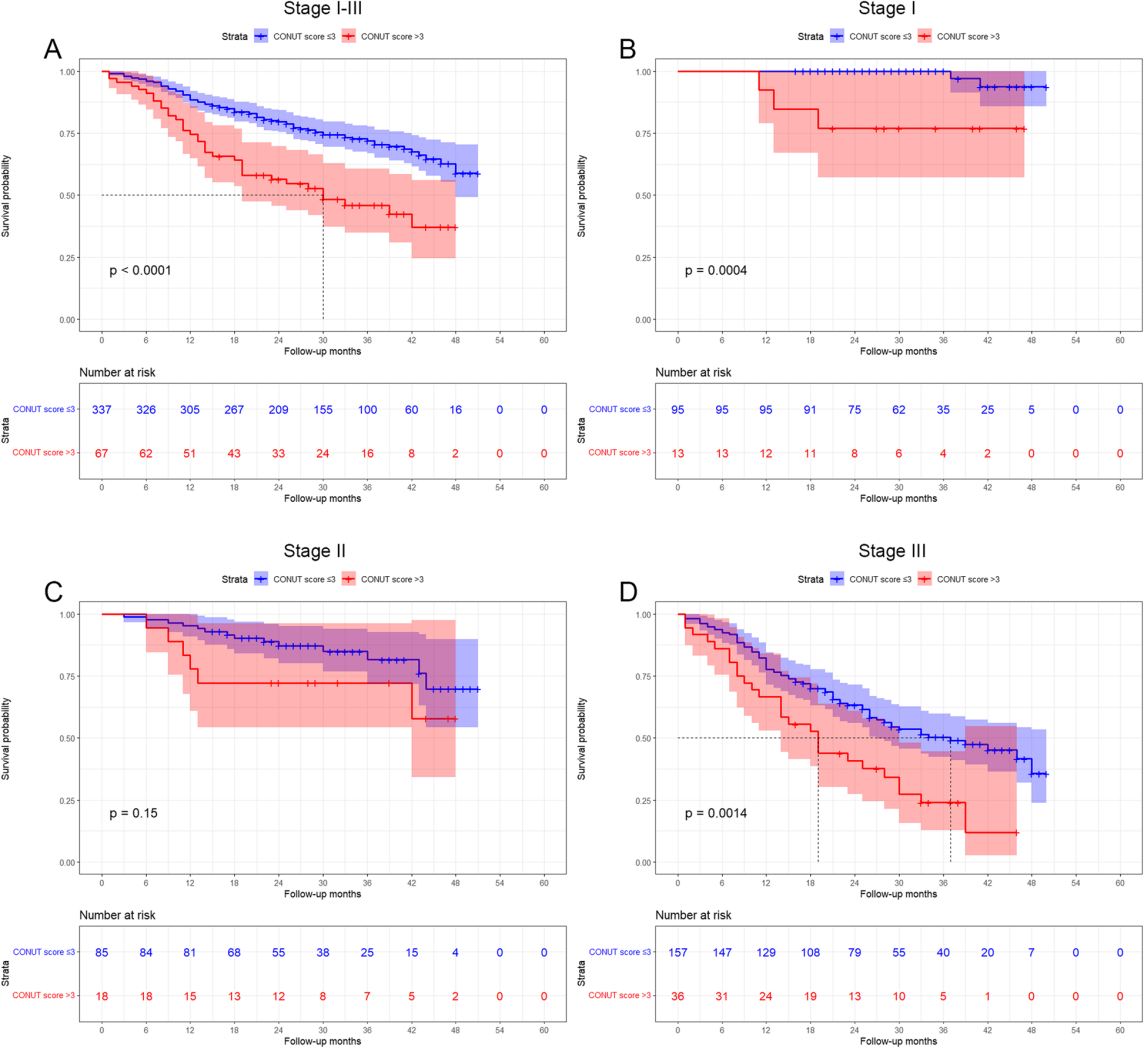
Kaplan–Meier analysis of OS at each pTNM stage according to CONUT score

Age, BMI, HGB, Serum ALB, Serum CEA, CONUT score, tumor size, pT stage, pN stage, nerve invasion, vascular invasion, tumor differentiation, and postoperative chemotherapy were the factors that influenced the prognosis of GC, according to the Cox's univariate analysis (*P* < 0.05 for all). The variables that independently predicted OS for GC patients were the age (*P* = 0.015), CONUT score (*P* = 0.002), pT stage (T3 vs T1: *P* = 0.011, T4 vs T1: *P* = 0.026), pN stage (N2 vs N0: *P* = 0.002, N3 vs N0: *P* < 0.001), nerve invasion (*P* = 0.021) (Table 4). The Kaplan–Meier analysis demonstrated a significant association between a high CONUT score and a shorter OS (*P* < 0.001, Fig. 1A). In order to undertake a more detailed examination of the performance of the CONUT score in patients with varying pTNM stages, we carried out a subgroup analysis. When stratified according to pTNM stage, there was no statistically significant disparity in OS between the high CONUT score group and low CONUT score group of patients with stage II GC (*P* = 0.151; Fig. 1C). Nevertheless, the predictive significance of the CONUT score remained significant for stage I (*P* = 0.001; Fig. 1B) and stage III (*P* < 0.001; Fig. 1D).

**Table 4:** Cox regression analysis of the factors influencing GC patients' prognoses

Variables	Univariate analysis			Multivariate analysis		
	HR	CI	*p*	HR	CI	*p*
Age (> 60 vs ≤ 60 years)	1.704	1.022–2.841	0.041	1.974	1.143–3.409	0.015
Sex (male vs female)	1.433	0.942–2.181	0.093			
Smoking (yes vs no)	1.355	0.874–2.101	0.174			
Drinking (yes vs no)	0.783	0.456–1.344	0.375			
Hypertension (yes vs no)	1.199	0.830–1.733	0.333			
Diabetes (yes vs no)	1.484	0.864–2.549	0.153			
COPD (yes vs no)	1.652	0.838–3.258	0.147			
Previous abdominal surgery (yes vs no)	0.832	0.499–1.387	0.481			
BMI			0.071			0.346
18.5–23.9 vs ≥ 24.0 kg/m^2^	1.443	0.915–2.277	0.115	1.143	0.708–1.844	0.585
< 18.5 vs ≥ 24.0 kg/m2	1.964	1.101–3.504	0.022	1.554	0.838–2.882	0.162
HGB (< 110/120 vs ≥ 110/120 g/L)	1.616	1.141–2.289	0.007	0.878	0.578–1.335	0.543
ALB (< 35 vs ≥ 35 g/L)	1.738	1.088–2.777	0.021	0.734	0.373–1.447	0.372
CONUT score	1.213	1.120–1.313	< 0.001	1.227	1.075–1.400	0.002
Serum CEA (> 5 vs ≤ 5 ng/mL)	1.664	1.143–2.423	0.008	1.064	0.710–1.596	0.763
Serum CA125 (> 35 vs ≤ 35 U/mL)	0.914	0.291–2.877	0.879			
Serum CA199 (> 39 vs ≤ 39 U/mL)	1.349	0.844–2.157	0.211			
Laparoscopy-assisted (yes vs no)	1.380	0.973–1.956	0.071			
Tumor location			0.345			
Middle vs Upper	0.942	0.607–1.463	0.791			
Lower vs Upper	0.738	0.488–1.116	0.150			
Type of reconstruction			0.463			
Billroth I vs Roux-en-Y	0.318	0.044–2.279	0.254			
Billroth II vs Roux-en-Y	0.836	0.424–1.649	0.606			
Combined resection (yes vs no)	1.127	0.676–1.880	0.646			
Tumor size (> 4 vs ≤ 4 cm)	2.475	1.701–3.602	< 0.001	0.838	0.548–1.279	0.412
p T stage			< 0.001			0.006
T2 vs T1	0.849	0.155–4.634	0.850	1.720	0.197–15.024	0.624
T3 vs T1	11.719	4.214–32.595	< 0.001	16.485	1.886–144.121	0.011
T4 vs T1	11.718	4.282–32.066	< 0.001	11.794	1.342–103.655	0.026
p N stage			< 0.001			< 0.001
N1 vs N0	1.415	0.686–2.919	0.347	0.981	0.455–2.116	0.962
N2 vs N0	4.394	2.582–7.477	< 0.001	2.689	1.458–4.959	0.002
N3 vs N0	7.422	4.523–12.178	< 0.001	4.715	2.561–8.680	< 0.001
Nerve invasion (yes vs no)	3.573	2.424–5.268	< 0.001	1.735	1.088–2.768	0.021
Vascular invasion (yes vs no)	2.731	1.893–3.939	< 0.001	0.676	0.424–1.077	0.100
Borrmann type			0.189			
II vs I	1.195	0.578–2.470	0.630			
III vs I	1.460	0.612–3.487	0.394			
IV vs I	2.021	0.878–4.653	0.098			
Tumor differentiation			0.005			0.534
Moderate vs well	11.555	1.537–86.863	0.017	3.589	0.383–33.593	0.263
Poor vs well	17.871	2.495–128.024	0.004	3.370	0.366–31.039	0.284
Postoperative chemotherapy (yes vs no)	6.775	2.502–18.343	< 0.001	0.199	0.023–1.752	0.146

### Development and assessment of the nomogram

**Fig. 2:**
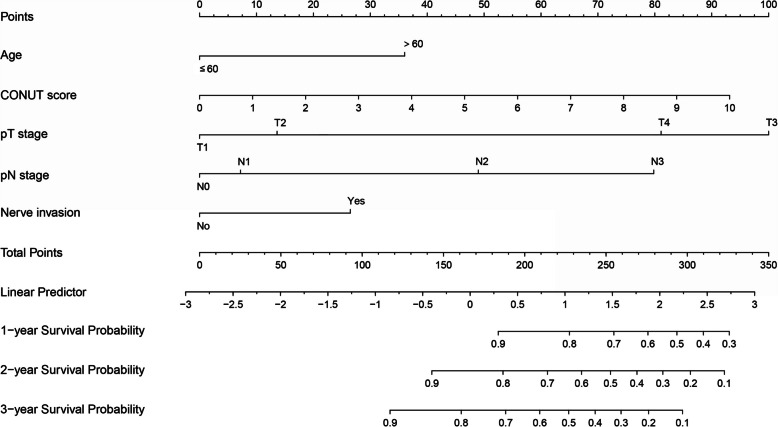
Nomogram for predicting GC patients' post-surgery prognosis

**Fig. 3:**
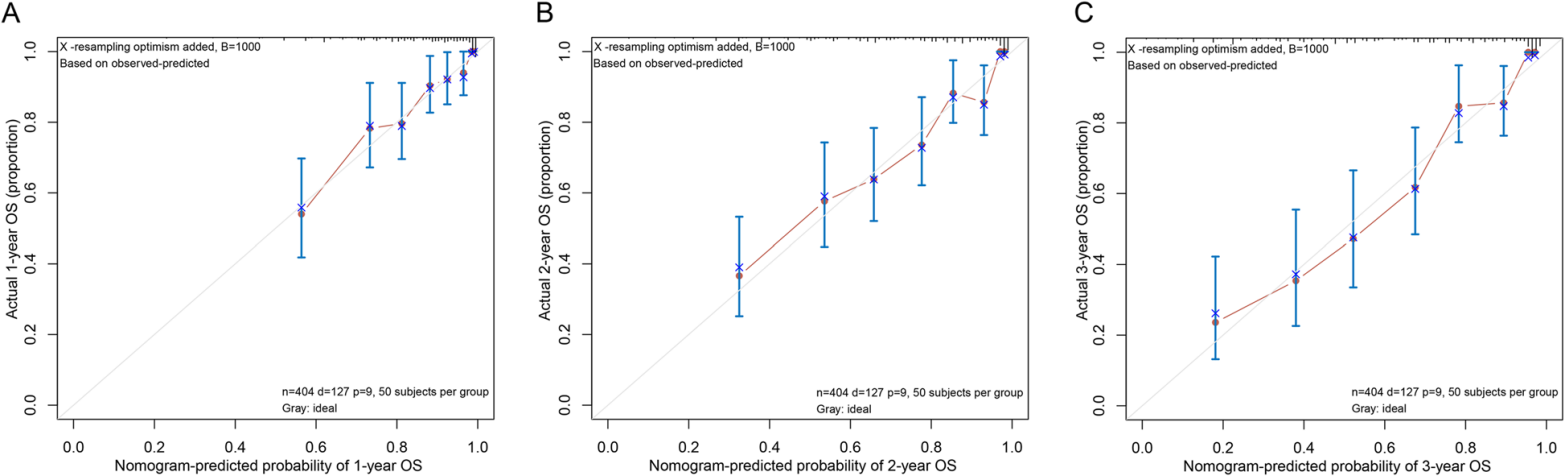
The calibration curves for predicting 1-year (**A**), 2-year (**B**), and 3-year (**C**) OS

Based on the outcomes of multivariate Cox regression analysis, a nomogram containing age, CONUT score, pT stage, pN stage and nerve invasion was created for predicting the prognosis of GC patients (Fig. 2). From the weighted total score derived for each variable, the nomogram could determine the corresponding 1-, 2-, and 3-year OS. A lower overall score improves the clinical prognosis. Prediction made by Nomogram most closely resembled the actual results, as seen by the calibration curve of postoperative 1-year, 2-year, and 3-year survival. (Fig. 3A, B, C).

### Performances of the nomogram

**Fig. 4:**
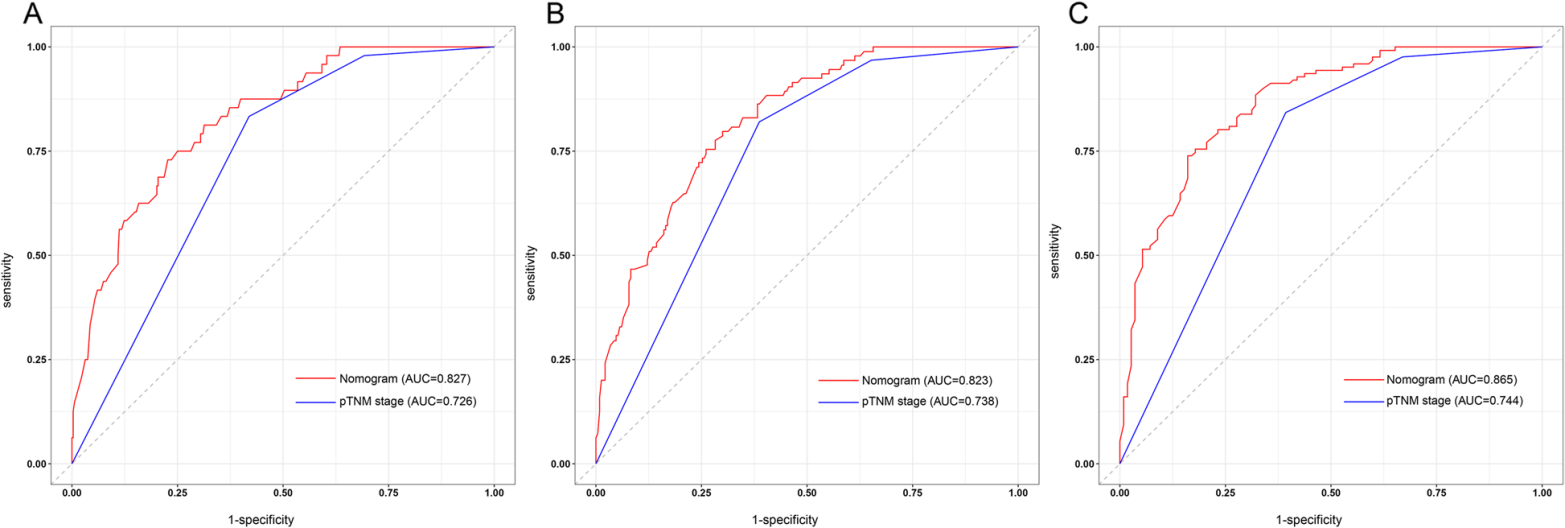
The ROC curves of nomogram and pTNM stage for predicting1-year (**A**), 2-year (**B**), and 3-year (**C**) OS

**Fig. 5:**
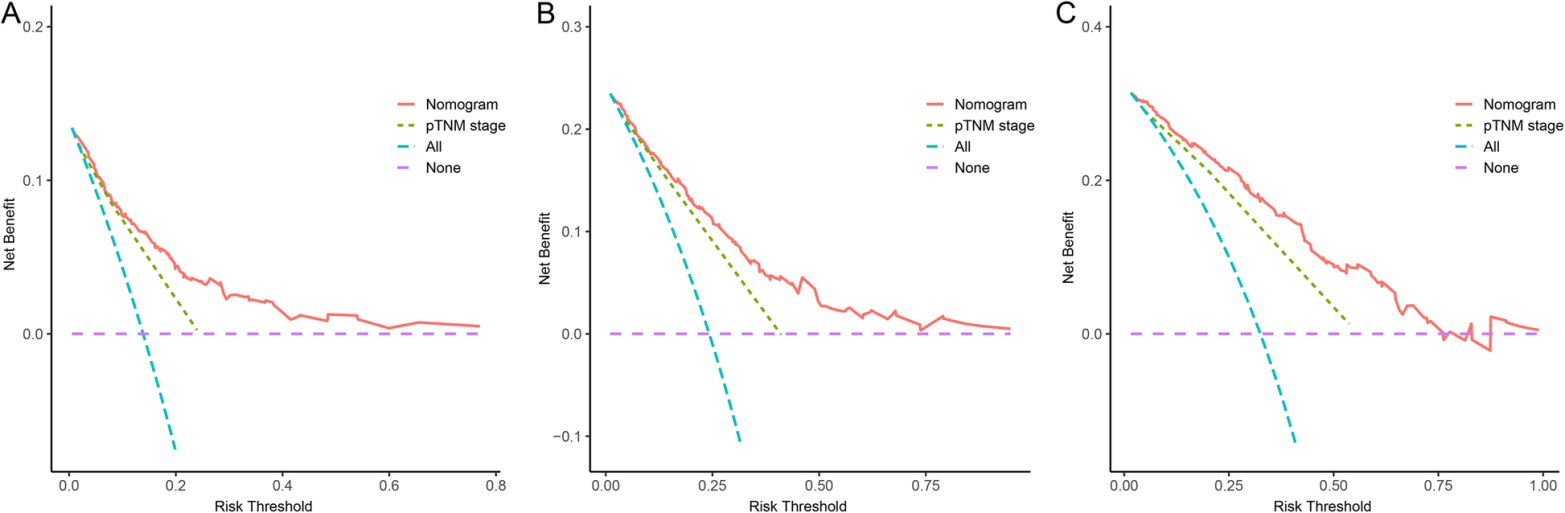
The decision curve analysis of nomogram and pTNM stage for predicting 1-year (**A**), 2-year (**B**), and 3-year (**C**) OS

The AUC and C-index were employed to measure the predictive ability and discrimination of the nomogram model. The 1-, 2-, and 3-year AUCs for OS predicted by the nomogram containing the CONUT score were 0.827, 0.823, and 0.865, respectively, whereas the AUCs for OS predicted by using the pTNM stage alone were 0.726, 0.738, and 0.744, respectively (Fig. 4A, B, C). The C-index for the nomogram model and pTNM stage to predict OS was 0.792 (95% CI: 0.755–0.829) and 0.718 (95% CI: 0.683–0.753), respectively. The clinical utility of the nomogram and pTNM stage was assessed using decision curve analysis. The decision curve analysis demonstrates that the nomogram exhibited a positive net benefit throughout a broad spectrum of decision threshold probabilities (Fig. 5A, B, C).

## Discussion

One of the most prevalent malignant tumors of the digestive system, GC, has a high incidence and fatality rate globally [1]. The prognosis for GC has yet to be much enhanced despite the tremendous advancements in treatment approaches involving surgical techniques and adjuvant chemotherapy [11]. To maximize postoperative individualized care, it is crucial to establish independent prognostic markers. A nomogram is a helpful tool that, by using easily accessible clinical data and advanced statistical analysis, gives oncologists and patients straightforward, understandable prognostic information. Numerous studies have shown that the nomogram model, which combines multiple influencing elements, outperforms the TNM stage in forecasting the individual survival of GC patients [4, 12–14].

This study's multivariate analysis suggested that age, CONUT score, pT stage, pN stage, and nerve invasion were independent predictor variables of prognosis in individuals having a radical resection for GC. A nomogram model was subsequently created utilizing the risk factors described above, and the nomogram model containing the CONUT score enhanced the ability to predict OS in GC patients using the pTNM stage alone. As far as we know, this work initially offers a nomogram model based on the CONUT score and clinical parameters to predict the prognosis for resectable GC patients.

Because it enables the prediction of therapy tolerance and cancer development, the nutritional condition of the GC patient is an essential component [15]. tumor invasion is associated with poor nutritional status, which may reflect metabolic increase brought on by the disease, immunological compromise brought on by tumor development, and sensitivity to cancer therapy [16, 17]. Additionally, it has been reported that the immune state of cancer patients influences their prognosis. Blood counts for neutrophils, lymphocytes, monocytes, and platelets have been shown to reflect both local and systemic inflammation connected to the progression and prognosis of cancer [18–20].

To improve the postoperative prognosis of GC patients, it is essential to identify and concentrate on modifiable risk factors and address them accordingly [21]. The patient's nutritional state is one of many factors influencing the prognosis of patients with tumors that can be modified, in contrast to non-modifiable factors like age, comorbidities, and tumor stage. It is necessary to conduct a comprehensive nutritional evaluation to detect and provide treatment for individuals experiencing malnutrition or those at high risk of malnutrition. The CONUT score is an objective evaluation tool that can be determined preoperatively and relies solely on laboratory measurements to mirror protein reserves, immune system functions, and lipid metabolism. ALB is related to the patient's nutritional status, tumor cells' generation of inflammatory cytokines, and liver function reserve [22]. Lymphocytes are crucial biological elements of the immune system in humans that help to stop the spread, assault, and migration of cancer cells [23]. Total cholesterol, an essential part of cell membranes, participates in several signalling pathways connected to tumor pathogenesis and development [24]. A multivariate study by Qian Y et al. demonstrated that the preoperative CONUT score was a risk factor that may independently predict postoperative complications [6]. Kuroda D et al. discovered from univariate and multivariate studies that patients with CONUT-high GC had significantly lower OS than patients with CONUT-low GC [7]. According to Liu's study's findings, which involved 697 consecutive patients who underwent curative surgery for Stage II-III GC, the 5-year cancer-specific survival rate was significantly lower in the high CONUT group compared with the low CONUT group [25]. Our findings further support the independent predictive utility of patients with high CONUT scores for GC patients. Therefore, the CONUT score offers a comprehensive, rapid, and efficient nutritional assessment approach for determining a cancer patient's prognosis.

The nomogram plays an essential part in the personalization of oncological therapy by combining several predictive variables to calculate the likelihood of clinical outcomes [26]. In the current study, we created a nomogram incorporating the age, CONUT score, pT stage, pN stage, and nerve invasion to improve postoperative outcome prediction in GC patients. Interestingly, for postoperative OS in patients with gastric cancer, the nomogram model containing the CONUT score improved the predictive ability of the traditional pTNM stage alone. Moreover, the nomogram model containing the CONUT score showed a more extensive net benefit at 1, 2, and 3 years. Consequently, in clinical practice, GC patients with high preoperative CONUT scores should undergo an attempt at nutritional resuscitation and reduced postoperative follow-up intervals for the assessment of their nutritional status. Moreover, individuals with high postoperative scores may gain from postoperative nutritional treatment, considering the beneficial effects of targeted nutritional intervention[27, 28]. However, as of now, the optimal nutritional strategy to improve cancer-related malnutrition has yet to be identified. Given this, it is necessary to further evaluate the effectiveness of preoperative and postoperative nutritional support based on the CONUT score using a prospective randomized controlled study.

Although this study obtained some excellent results, there are still several restrictions. Firstly, in contrast to a multi-center survey, this study is a retrospective review of a single center, and therefore, we could not avoid potential biases. Secondly, due to the absence of external validation in the present research, the predictive utility of the CONUT score in GC patients needs to be validated in additional population studies. Finally, we needed more information on postoperative complications and various nutritional supplements; more research is necessary. Therefore, multi-center investigations and more extensive population studies are needed to corroborate our findings. Despite these drawbacks, our model helped predict the OS of GC patients and may be utilized to direct patients' individual treatment regimens.

## Conclusion

In conclusion, this study concluded that the prognostic nomogram constructed based on the CONUT score enhanced the predictive ability of the conventional TNM staging system alone for OS in patients with resectable GC. The prognostic nomogram based on the CONUT score is an objective and reliable tool for identifying patients with high prognostic risk and assisting the development of personalized strategies for resectable GC patients.

## Data Availability

The corresponding author will provide the datasets used and analyzed during the current work upon reasonable request.
